# A randomised clinical study to determine the effect of a toothpaste containing enzymes and proteins on plaque oral microbiome ecology

**DOI:** 10.1038/srep43344

**Published:** 2017-02-27

**Authors:** S. E. Adams, D. Arnold, B. Murphy, P. Carroll, A. K. Green, A. M. Smith, P. D. Marsh, T. Chen, R. E. Marriott, M. G. Brading

**Affiliations:** 1 Unilever R&D Port Sunlight, Bebington, Wirral, CH63 3JW, UK; 2 Unilever R&D Colworth Science Park, Sharnbrook, Bedfordshire, MK44 1LQ, UK; 3 School of Dentistry, University of Leeds, LS2 9LU, UK; 4 Forsyth Dental Institute, 245 First Street, Cambridge, MA 02142, USA

## Abstract

The numerous species that make up the oral microbiome are now understood to play a key role in establishment and maintenance of oral health. The ability to taxonomically identify community members at the species level is important to elucidating its diversity and association to health and disease. We report the overall ecological effects of using a toothpaste containing enzymes and proteins compared to a control toothpaste on the plaque microbiome. The results reported here demonstrate that a toothpaste containing enzymes and proteins can augment natural salivary defences to promote an overall community shift resulting in an increase in bacteria associated with gum health and a concomitant decrease in those associated with periodontal disease. Statistical analysis shows significant increases in 12 taxa associated with gum health including *Neisseria* spp. and a significant decrease in 10 taxa associated with periodontal disease including *Treponema* spp. The results demonstrate that a toothpaste containing enzymes and proteins can significantly shift the ecology of the oral microbiome (at species level) resulting in a community with a stronger association to health.

The human body’s resident microbiota is not only essential for life but also plays a critical role in both the protection from, and development of, various diseased states^1^. As described by Kilian *et al*., humans have co-evolved with microorganisms and have a symbiotic or mutualistic relationship with their resident microbiome which, for the most part, remains homeostatic^2^. However, the bacteria, viruses and fungi that occupy our body sites, be it the scalp^3^, the face^4^, the gut^5^ or the oral cavity^6^ have also been linked through causation or correlation, to a number of disease states and cosmetic conditions including dandruff^7^, acne^8^, inflammatory bowel disease^9^ and periodontitis^10^.

When in equilibrium in the oral cavity, the microbiota forms an ecosystem that is important in the promotion and maintenance of health^11^. The mouth harbours one of the most diverse microbiomes in the body which includes viruses, fungi, protozoa and archaea as well as bacteria^12^. Over 700 different microbial species have been identified to date^13^,^14^. While host predisposition to oral disease has been shown to be important^15^,^16^,^17^, in general, the presence of such a highly diverse microbial community prevents outgrowth of any single species which otherwise might lead to a bacterial load exceeding the pathological threshold^18^. However, the benefits of the oral microbiota go beyond protection from colonisation by exogenous microbes and include immunological priming and down-regulation of excessive pro-inflammatory responses^19^. The symbiotic relationship can break down, for example, through poor oral health regimes, resulting in dysbiosis and plaque-related diseases^20^.

Saliva plays an important role in preventing dysbiosis and maintaining health in the oral cavity^21^,^22^. Salivary components, particularly antimicrobial factors such as enzymes and proteins, exert significant selective pressures on the microbiota, helping to provide protection against pathogenic organisms^23^,^24^ and helping to shape and control the resident community^25^. Saliva is also important in the formation of the pellicle, the thin acellular organic film that forms on oral surfaces following exposure to saliva^26^. Salivary enzymes and proteins have been shown to be incorporated into the salivary pellicle^27^, to be immobilised in an active form at the surface^28^ and to directly influence the pattern of initial microbial colonisation^29^.

One of the main defence mechanisms of saliva is the lactoperoxidase system (LPO system)^28^. This system is activated in part by hydrogen peroxide which oxidises thiocyanate to hypothiocyanite, a process that has been discussed extensively in the literature^30^,^31^,^32^. Hydrogen peroxide itself possesses antimicrobial activity^33^ and has been shown to play an important role in oral health. Hydrogen peroxide in the oral cavity is produced by both the human host^34^ and members of the oral microbiome. Levels of hydrogen peroxide produced by some streptococcal species in the laboratory have been described as being sufficient to inhibit the growth of many plaque bacteria^35^. Hydrogen peroxide also participates in a number of enzymatic reactions in addition to the LPO system, including the production of oxygen by the enzyme catalase^23^. In addition, hypothiocyanite plays a key role in oral health as a natural antimicrobial and has been extensively researched^34^,^36^,^37^,^38^,^39^,^40^. Hypothiocyanite produced by the LPO system has antibacterial effects on both cariogenic bacteria^32^ and black pigmented anaerobic bacteria associated with periodontal disease such as *Porphyromas gingivalis*^41^.

In addition to the LPO system, other salivary components including lysozyme and lactoferrin are critical to the mouth’s natural defences against bacteria. Lysozyme is an antibacterial protein found in a variety of mucosal fluids^18^. Quantitatively, it is the most important salivary component with antibacterial properties, due to its ability to break glycosidic linkages in peptidoglycans^21^,^42^. The effect is most pronounced against Gram-positive bacteria due to the thick peptidoglycan layer in the cell wall, whereas the peptidoglycan is protected by an outer membrane in Gram-negative bacteria^43^. Lactoferrin has been shown to permeabilise the outer membrane of Gram-negative bacteria making them susceptible to penetration by lysozyme^44^. This action of lactoferrin is in addition to its main mode of action as an iron-binding protein which reduces the concentration of iron available as a co-factor for bacterial enzymes and in turn retards bacterial growth. As well as bacteriostatic properties, lactoferrin is known to have bactericidal properties in its own right resulting from direct interaction between the protein and bacteria^45^. Lactoferrin and lysozyme have been shown to work synergistically in combination^46^ and additionally both lactoferrin and lysozyme have been reported to have elevated antimicrobial activity when combined with the LPO system^39^,^47^. Furthermore lactoferrin has been linked to anti-inflammatory activity against periodontitis^48^.

To boost the role of natural salivary defences in controlling the oral microbial community, oral hygiene products including toothpastes have been developed that contain enzymes and proteins. Zendium™ contains a three enzyme system (amyloglucosidase, glucose oxidase and lactoperoxidase), designed to promote the generation of hydrogen peroxide and hypothiocyanite, as well as three further protein components (lysozyme, lactoferrin and immunoglobulin IgG), designed to provide additional antimicrobial benefits. Both lysozyme and hydrogen peroxide have been shown to be elevated in saliva after brushing with Zendium™ compared to a control toothpaste without the enzymes and proteins^49^. This is of significance as it has been reported in the literature that normal physiological levels of hydrogen peroxide may be too low to activate the LPO system^31^ and enhancement of the LPO system *in vivo* may be effective in the regulation of acid producing bacteria^50^.

Studies reporting the effect of toothpastes on the ecology of the oral microbiome, have for the most part, been limited to the use of traditional culture based methods^51^,^52^. This has limited our understanding as a large proportion of the resident microbiota cannot be grown in the laboratory^53^. Despite the rapidly emerging use of molecular techniques, microbial ecology studies reporting changes in the oral microbiome after toothpaste use are currently sparse in the scientific literature^54^,^55^. With the latest developments in DNA sequencing technology, it is possible to measure community level changes in the oral microbiome, highlighted by the wealth of recent studies investigating the differences between healthy and diseased states^16^,^56^,^57^. These studies have been facilitated by the availability of bespoke, highly curated databases that allow the assessment of human associated microbiomes to species level e.g Human Oral Microbiome Database^58^,^59^.

Given the complexity of the microbial community it is essential to make an assessment at the species level to explore the contribution of individual species to the overall community function. The objective of this work was to understand the effect of toothpaste use on the ecology of the oral microbiome at the species level, comparing a fluoride toothpaste containing enzymes and proteins with a fluoride toothpaste without enzymes and proteins. Any changes observed provide insights into the benefits of using a toothpaste with enzymes and proteins to boost natural salivary defences, shift oral ecology and provide potential health benefits.

## Results

### Sequence processing and taxonomic classification

Two hundred and twenty samples were processed and analysed via Illumina sequencing, initially resulting in approximately 37.9 million raw sequence paired reads which, following quality processing, produced 26.9 million overlapping contigs. 14.7 million contigs were successfully classified to genus/species level following use of The Forsyth Institute pipeline resulting in 17 phyla, 183 genera and 1220 species level taxa. Taxa with counts of fewer than 100 reads were aggregated; leaving 414 species level taxa taken forward for statistical analysis. Eight paired samples were removed at this stage due to either generation of no sequence data (4 samples) or fewer than 20,000 reads (4 samples). The remaining 204 samples were processed through the statistical analysis pipeline.

### Community changes – Genus level

**Figure 1:**
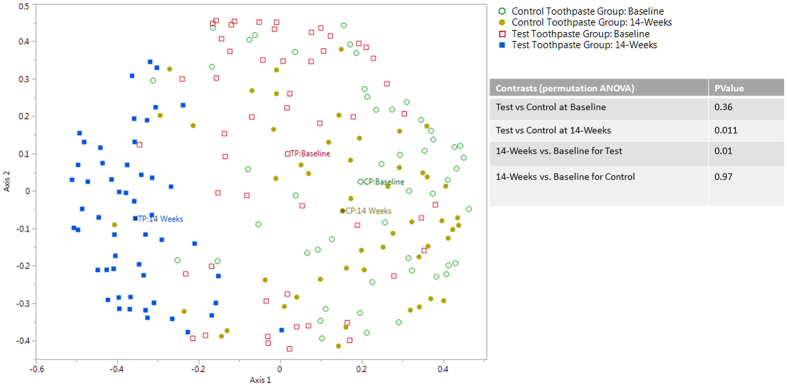
Genus level: Ordination plot showing results of random forest analysis for genus level data for the four experimental groups. The label indicates the centroid for each experimental group.

Analysis was carried out at genus level to determine the genera affected by use of the toothpastes over 14-weeks. Beta diversity was used to examine the differences between sample groups and visualised using ordination plots. The ordination plot of the random forest analysis (Fig. 1) shows the bacterial communities for both toothpastes at the baseline and 14-week time points. ANOVA was used to compare the two toothpaste groups. No significant difference was observed between the bacterial communities at baseline (p = 0.36). The data was assessed for community changes over the 14-week study period and this highlighted a significant shift in the community profile for the test toothpaste users (p = 0.01) but no such shift for control toothpaste users (p = 0.97). A significant difference between the bacterial communities was observed between both toothpaste groups at 14-weeks (p = 0.011).

### Community changes – Species level

**Figure 2:**
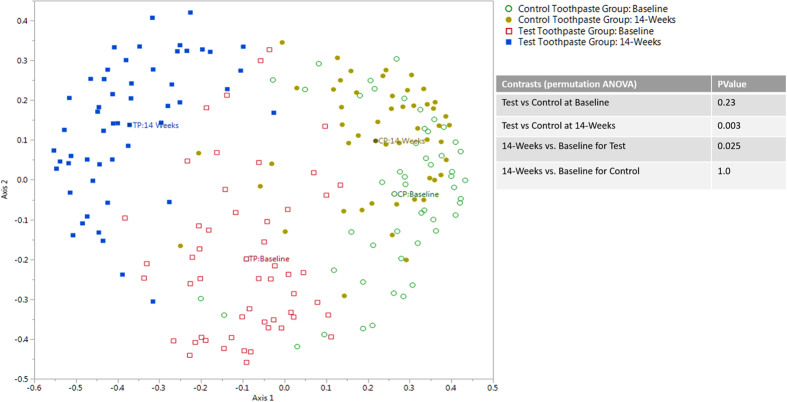
Species level: Ordination plot showing results of random forest analysis for species level data for the four experimental groups. The label indicates the centroid for each experimental group.

The outcome of the analysis at the species level was consistent with the genus level results. ANOVA and associated ordination plots of the random forest analysis (Fig. 2) showed no significant difference in communities at baseline (p = 0.23) while significant community shifts were observed for the test toothpaste users over 14-weeks (p = 0.025). No differences were observed for control toothpaste users (p = 1.0). A statistically significant difference was observed between the test and control toothpastes at the 14-week time point (p = 0.003).

**Figure 3:**
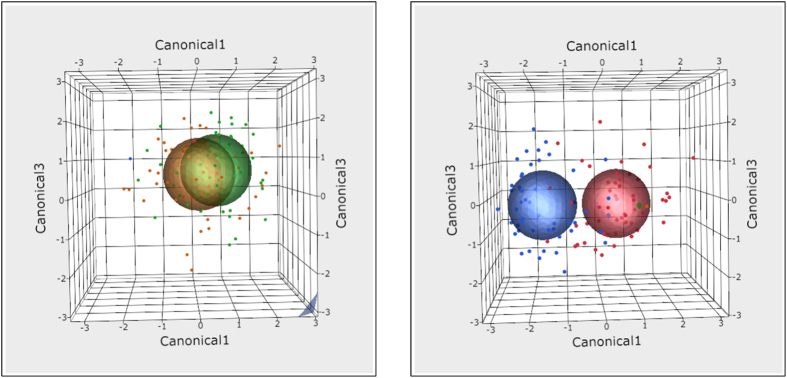
Three dimensional ordination plots showing the 95% confidence ellipses for each toothpaste and time point at species level. Green – Control Toothpaste Group at Baseline, Yellow – Control Toothpaste Group at 14-weeks, Red – Test Toothpaste Group at Baseline, Blue – Test Toothpaste Group at 14-weeks.

**Figure 4:**
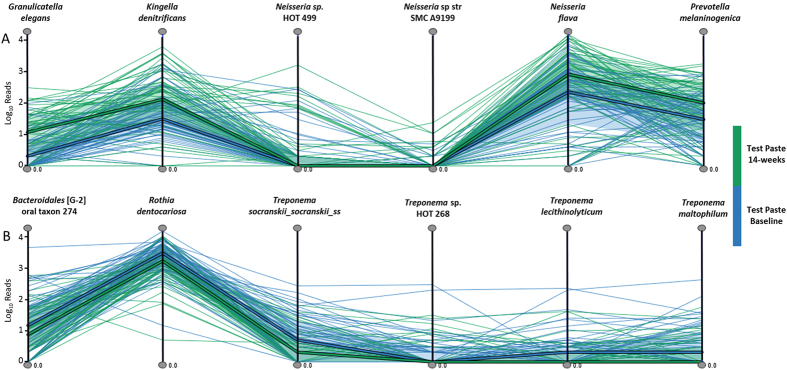
MicrobiVis visualisation examining the distribution of counts for selected species observed to have (**A**) increased or (**B**) decreased in relative abundance following the use of the test toothpaste over the 14-week time period. The vertical lines represent the named taxa with the thin lines representing the logarithmic abundance for each sample for the different taxa at baseline (blue) and at 14-weeks (green). The thick line represents the median level for each taxon and the shaded area the interquartile range.

Whilst representing data in two dimensions is informative, visualising these data in three dimensions provided a better fit to the spatial distribution. The three dimensional model differentiated the sample groups providing an easy to interpret exploratory visualisation (Fig. 3). MicrobiVis was used to visualise changes in relative abundance of selected species (Fig. 4). Visualisation carried out in this way allowed assessment of the distribution of taxa between individual samples.

### Analysis of Species Changes

To understand in more detail, the in-group and between-group differences observed through ordination and ANOVA measures, the mean relative abundance of individual species was compared between toothpastes over time. The 414 species were examined for changes in relative abundance from baseline to 14-weeks, for each toothpaste, using Dirichlet Multinomial algorithm^60^. The positive false discovery rate was tightly controlled to within 5% using the q-value. The q-value is an adjusted p-value method^61^. This allowed the identification of statistically significant taxa changes with q < 0.05.

**Figure 5:**
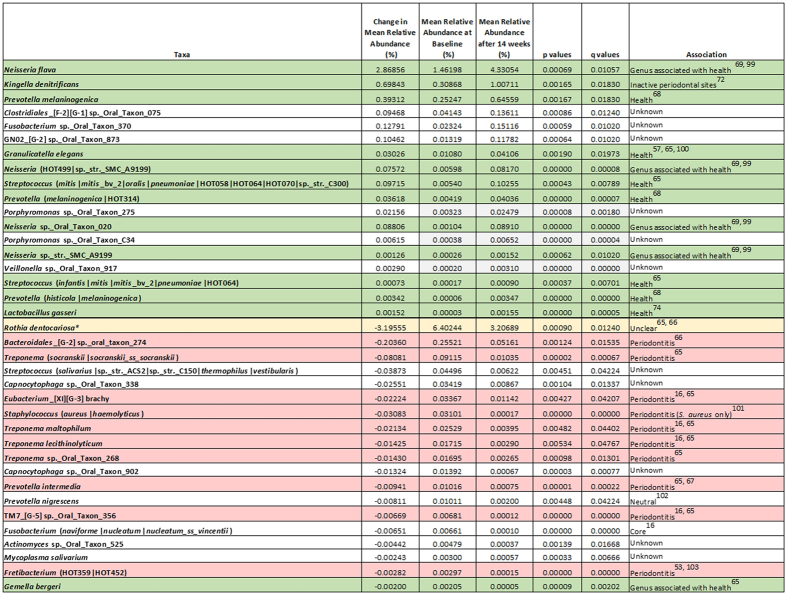
Summary of the significant species changes associated with gum health and/or disease for the Test toothpaste after 14-week test period. *Rothia dentocariosa has been reported in the literature to be associated with both health and periodontal disease.

**Figure 6:**
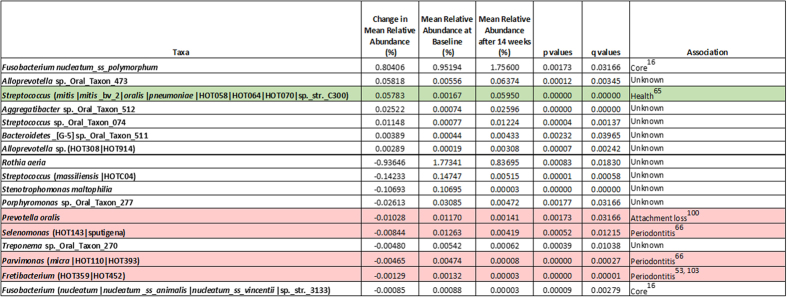
Summary of the significant species changes associated with gum health or disease for the Control toothpaste after 14-week test period.

Significant changes in the abundance of 54 taxa were observed, 37 for the test toothpaste users and seventeen for control toothpaste users (Figs 5 and 6). Two taxa were identified as having changed in abundance for both toothpastes; one taxon consisted of members of the mitis group streptococci and the other a *Fretibacterium* sp. For each toothpaste, significant taxa were assessed in terms of increase or decrease in mean relative abundance expressed as a percentage of the total community. For the test toothpaste group, 18 taxa increased in relative abundance and 19 decreased in relative abundance. For the control toothpaste group, 7 taxa increased in relative abundance and 10 decreased in relative abundance.

The species with the largest increase in relative abundance in the test toothpaste group after 14-weeks of use was *Neisseria flava* with a 2.9% change. Three other *Neisseria* species also increased in relative abundance. No significant changes were observed in *Neisseria* species for the control toothpaste where *Fusobacterium nucleatum* ss polymorphum showed the largest increase at 0.8%. The species with the largest decrease in relative abundance in the test toothpaste group after 14-weeks was *Rothia dentocariosa* with a 3.2% change. Four taxa from the genus *Treponema* were also found to decrease in relative abundance for the test toothpaste. In the control group, the largest decrease in relative abundance was also attributed to the *Rothia* genus but in this instance, *Rothia aeria* was identified, with a decrease of 0.9%.

## Discussion

In this paper we report for the first time an *in vivo* study using molecular metataxonomics^62^ that demonstrates species level changes in the ecology of the oral microbiome after toothpaste use. Specifically, we demonstrate that brushing with a toothpaste containing enzymes and proteins, Zendium™, promotes a shift in the ecology of the oral microbial community over time, compared to a toothpaste without enzymes and proteins in healthy subjects. This finding was consistent at both genus and species level and was informed by both state-of-the-art informatics processing and robust statistical analysis, without the need for rarefaction.

Importantly, the results confirm that a comparison of communities at the genus level, whilst informative, is insufficient to robustly discriminate the role of specific community members in driving the ecological shift. Within a bacterial genus, closely related taxa can provide different functionality within a community and as such could be drivers for health or disease. Therefore, reliable identification of the species provides an understanding of the potential for differential community impact. For example in the genus *Porphyromonas, P. gingivalis*, is associated with periodontal disease^63^,^64^, whilst *P. catoniae* is associated with health^16^. A review of the literature facilitated the association of the identified taxa with gum health and/or disease (Figs 5 and 6). It was shown that there was an increase in the relative abundance of organisms associated with gum health for the test toothpaste and a concomitant decrease in those organisms associated with periodontal disease.

For the test toothpaste group 12 taxa associated with health increased in relative abundance. 10 taxa associated with periodontal disease decreased in relative abundance. Of the remaining taxa, 11 currently have no known association with gum health or disease (Fig. 5). One taxon, *Rothia dentocariosa,* has been associated with both health and disease^65^,^66^.

For the control toothpaste group 1 taxon associated with health increased in relative abundance. 4 taxa associated with periodontal disease decreased in relative abundance. Of the remaining taxa, 10 currently have no known association with health or disease (Fig. 6). The single taxon associated with health that increased in the control group (mitis group streptococci) also increased in the test toothpaste group and the increase seen in the test toothpaste group was almost double (0.097%) the change in abundance observed in the control toothpaste group (0.057%).

### Species increased in relative abundance after test toothpaste use

Analysis at species level is critical to understand if changes within the microbiome are associated with health or disease. Justification of this approach is provided by analysis of significant changes in the relative abundance of the genus *Prevotella*. Some members of the genus *Prevotella*, such as *Prevotella intermedia* are associated with disease^67^. In this study we identified that *Prevotella melaninogenica* and a number of associated phylotypes increased in relative abundance. Importantly *P. melaninogenica* is an organism present more commonly in healthy plaque^68^. This observation further emphasises the necessity of classifying to species level to gain accurate information on the biological significance of the ecological changes occurring within communities.

Changes observed at species level are consistent with the mode of action of the enzymes and proteins within the Zendium toothpaste. For example*, Neisseria* species, commensal organisms representing some of the only aerobes in the mouth and generally associated with health^69^, increased in relative abundance. *Neisseria* species exhibit enhanced growth in an aerated mixed culture^70^ and as a catalase positive species^71^ they are able to protect themselves from the antimicrobial action of hydrogen peroxide subsequently raising local oxygen levels. We propose that this will give *Neisseria* a competitive advantage over anaerobic catalase negative organisms. The second most significant increase in relative abundance is attributed to *Kingella denitrificans*. Whilst little information exists in the literature, its taxonomic classification is in the same grouping as *Neisseria* suggesting that it occupies a similar ecological niche and would be likely to increase under the same conditions that promote the growth of *Neisseria spp*.. *K. denitrificans* has previously been shown to be increased in relative abundance in stable periodontal sites as opposed to active periodontal sites^72^, although all subjects in this study were healthy and free of periodontitis.

Other organisms shown to increase in relative abundance in the test toothpaste group after 14-weeks include *Granulicatella elegans. G. elegans* has been shown to be statistically more abundant in healthy sites from periodontally healthy individuals compared to periodontitis patients^73^. Indeed, Lourenco *et al*. proposed that the absence of *G. elegans* was associated with a higher risk of generalised, aggressive periodontitis in relation to chronic periodontitis. *Lactobacillus gasseri*, which has also been shown to increase in the test toothpaste group, has been screened recently for its potential use as a probiotic, having been shown in the laboratory to have antibacterial activity against *P. gingivalis* (ATCC 33277)^74^.

### Species decreased in relative abundance after test toothpaste use

Changes observed are consistent with the mode of action of the test toothpaste. As part of the enzyme cascade that leads to hypothiocyanite formation, hydrogen peroxide is also produced and increased levels of hydrogen peroxide have been observed *in-vivo*^49^ which in turn could lead to an increase in oxygen concentration through, for example, catalase activity. Such changes in the local environment would be expected to inhibit the growth of anaerobic species, and a number of obligatory anaerobic species were reduced (e.g. representatives of the genera *Treponema, Bacteroidales, Eubacterium, Prevotella, Fusobacterium* and *Fretibacterium*: Fig. 5) in those using the test toothpaste. *Treponema* species have been shown to be more abundant in periodontal disease^16^,^65^ and are known to be highly sensitive to oxygen^75^.

The largest decrease in relative abundance for the test toothpaste group was attributed to *Rothia dentocariosa*. Whilst there is no conclusive proof to this organism’s role in health or disease, as it has been shown to be associated with periodontal diseases^66^,^76^,^77^ as well as a positively associated with gum health^16^,^65^,^78^. This organism is a catalase positive aerobe^79^, occupying a similar niche to *Neisseria* spp so it is unlikely that the decrease in *Rothia* abundance can be attributed to an increase in hydrogen peroxide levels or increased oxygen concentration. However, *R. dentocariosa* is a Gram-positive bacterium and as such would be expected to be more susceptible to lysozyme, the levels of which are almost doubled after brushing with the test toothpaste^49^. This provides a rationale for the observed decrease in this organism’s relative abundance in the test toothpaste group. Whilst the role of *R. dentocariosa* remains undefined, other organisms known to be associated with periodontal disease also showed a decrease in the test toothpaste group. These included *Bacteroidales* [G-2] sp._oral_taxon_274, *P. intermedia* and *Eubacterium* [XI][G-3] *brachy.*

The data presented here confirm that a toothpaste formulated to augment the natural defences of saliva leads to a positive shift to a microbiome more associated with health. Specifically, it shows that a toothpaste containing enzymes and proteins, Zendium^TM^, can significantly increase the relative abundance of health-associated organisms in plaque whilst driving a concomitant decrease in a number of disease-associated organisms compared with a toothpaste without enzymes and proteins over time. In this healthy study population, the magnitude of the statistically significant changes in the abundance of some of the individual taxa appear relatively modest, however, the cumulative ecological effect of many small but beneficial shifts in health and disease associated species within the microbiome is hypothesised to have biological relevance. There is emerging evidence in the literature that differences in health status can be associated with modest compositional differences in microbial communities^80^. We propose that the regular use of a toothpaste containing enzymes and proteins can actively maintain a balanced microbiome, with a composition that is consistent with oral health, thereby helping to reduce the risk of dysbiotic changes associated with disease. This study was performed with subjects in good oral health over 14-weeks and it is possible that more extensive taxonomical and/or percentage abundance shifts in the microbiome could be achieved when the product is used for longer periods or in subjects with a greater risk of disease or poorer oral health.

This research provides new insights into both the action and microbial re-profiling abilities of this toothpaste. We demonstrate that an ecological approach using DNA sequencing, bespoke bacterial databases and rigorous statistical analysis provides a highly robust approach to assess the impact of oral care products on the ecology of the oral microbiome. Furthermore, brushing with Zendium^™^ exerts a significant positive shift in the plaque microbiome, which we propose provides evidence for the biological relevance of increased salivary defence factors in promoting oral health.

Further work in the area of metatranscriptomics analysis will provide functional profiling of microbial communities and will help determine the community metabolic output^72^,^81^,^82^. This, together with information on the spatial structure of the plaque biofilm community using techniques such as fluorescent *in situ* hybridisation^83^ will provide more detail as the overall effect of the beneficial changes reported here.

## Materials and Methods

### Ethics statement

Written informed consent was obtained from all enrolled individuals. The study protocol was reviewed and approved by the Unilever R&D Port Sunlight independent ethics committee. The methods were carried out in accordance with the approved guidelines.

### Participants

Subjects in good health aged 18 or over were recruited onto the study. The mean age of subjects was 42, with 33 male and 78 female participants completing the study. Demographic information for this study is given in Table S13 in the Supplementary Information. Key inclusion criteria included: minimum age 18 years, minimum number of teeth 20, no antibiotic therapy or professional cleaning within one month of the start of the study. Key exclusion criteria included: pregnancy, nursing mothers, diabetics, denture wearers, smoking within the last 6 months, medical conditions and/or regular use of any medication which might affect the outcome of the study and obvious signs of untreated caries/significant periodontal disease.

### Study Design

**Figure 7:**
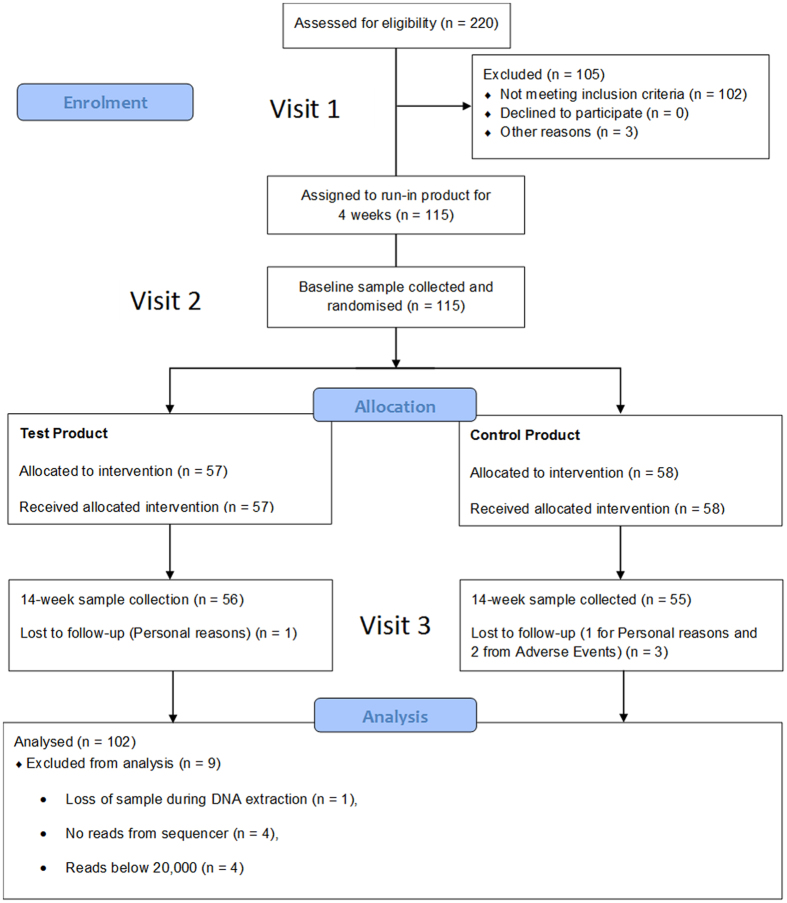
Study Flow Diagram.

This study was a double-blind, randomised, parallel group study conducted by an independent third party. A study flow diagram is shown in Fig. 7. Subjects were enrolled on to the study according to the inclusion/exclusion criteria. After recruitment, subjects were given a fluoride toothpaste to use for four weeks prior to the commencement of the study. Following the initial 4-week at home use, baseline supra-gingival plaque from the upper jaw was collected for the assessment of microbiome composition. Subjects were randomly allocated to one of two toothpastes, a fluoride toothpaste (1450 ppm) containing enzymes and proteins (Zendium™) or a control fluoride toothpaste (1450 ppm) without enzymes and proteins. Subjects were instructed to use the toothpaste at home, brushing twice a day for 14-weeks. Supra-gingival plaque from the upper jaw was collected at week 14 for the assessment of microbiome composition. Each plaque sample was placed in 1 ml TE buffer in a low-DNA-binding Eppendorf tube. The samples were stored at −25 °C until analysis.

### DNA extraction

Samples were defrosted, vortexed for 30 seconds in the original TE buffer, sonicated for 20 seconds and vortexed for a further 30 seconds. 500 μl of each sample was transferred into an individual well of a 96-well Lysis plate containing pre-aliquoted matrix beads B (116981001, MPBio, California, USA). 3 μl aliquots of Ready-Lyse lysozyme (Epicenter, Wisconsin, USA) were added and the plate incubated using an Eppendorf Thermomixer at 37 °C with shaking at 300 rpm for 18 hours. Following incubation, a bead-beating step was performed using a Tissue Lyser (Qiagen, Germany) for 3 minutes at 20 Hz. An off-board lysis was performed by incubating the samples at 68 °C for 15 minutes in the presence of Proteinase K, Carrier RNA, ATL and ACL buffer in a Qiagen S-plate following manufacturer guidelines. Post-incubation, the plate was loaded on to the QIAsymphony and the samples processed using the QIAsymphony Virus/Bact Midi Kit (931055, Qiagen).

DNA quantification was performed using a Quant-IT high sensitivity DNA Assay kit (Invitrogen, California, USA) following manufacturers guidelines. Each sample was normalised to 1 ng/μl using molecular grade water on a QIAgility robot (Qiagen) prior to PCR amplification.

### 16S rRNA gene amplicon library preparation

Oligonucleotide primers targeting the V4-V6 hypervariable region of the 16S rRNA gene were evaluated *in silico* using PrimerProspector^84^. PCRs were conducted using the primers listed below (underlined section of the primer denotes the PCR attachment sequence). Primers were modified from the standard 533f and 1061r to include recognition sequences allowing a secondary nested PCR process facilitating the addition of standard Illumina adapters and sample specific indexes.

533f 5′ACACTCTTTCCCTACACGACGCTCTTCCGATCTNNNNNGTGCCAGCMGCCGCGGTRA3′ and

1061r 5′GTGACTGGAGTTCAGACGTGTGCTCTTCCGATCTCRRCACGAGCTGACGAC3′.

PCRs consisted of 0.25 μl (10 μM) of each primer, 7 μl of HotStar Taq Plus Mastermix (Qiagen), 5 μl of normalized template DNA and 4.5 μl molecular grade water (Qiagen). Samples were amplified in triplicate using the following parameters: 95 °C for 5 minutes, then 10 cycles of: 94 °C for 45 seconds, 65 °C for 30 seconds, and 72 °C for 60 seconds, with a final extension of 10 minutes at 72 °C using a Biorad T100™ (Biorad, California, USA). Replicate PCR amplicons were pooled and sent to an external sequencing provider. Positive (mock community) controls were included in all PCR batches to highlight erroneous PCR amplification.

PCR products were purified using Axygen SPRI Beads (Axygen, California, USA). A second round PCR incorporated Illumina adapters containing indexes (i5 and i7) for sample identification utilising eight forward primers and twelve reverse primers each of which contained a separate barcode allowing up to 96 different combinations. General sequences of the primers are illustrated below with the variable 8 bp barcode underlined.

N501 f 5′AATGATACGGCGACCACCGAGATCTACAC*TAGATCGC*ACACTCTTTCCCTACACGACGCTC3′

N701 r 5′CAAGCAGAAGACGGCATACGAGAT*TCGCCTTA*GTGACTGGAGTTCAGACGTGTGCTC3′.

Second round PCRs consisted of 0.5 μl (10 μM) of each primer, 10 μl of 2 x Kapa Mastermix (Kapa Biosystems, Massachusetts) and 9 μl of purified sample from the first PCR reaction. Samples were amplified using the following parameters: 98 °C for 2 minutes, then 15 cycles of; 20 seconds at 95 °C, 15 seconds at 65 °C, 30 seconds at 70 °C with a final extension of 5 minutes at 72 °C. Samples were purified using Axygen SPRI Beads before being quantified using Qubit fluorimeter (Invitrogen, California, USA) and assessed using the Fragment Analyzer (Advanced Analytical Technologies, Iowa, USA). Resulting amplicon libraries were taken forward and pooled in equimolar amounts using the Qubit and Fragment Analyzer data and size selected on the Pippin prep (Sage Science, Massachusetts, USA) using a size range of 300–600 bps. The quantity and quality of each pool was assessed by Bioanalyzer (Agilent Technologies, California, USA) and subsequently by qPCR using the Illumina Library Quantification Kit (Kapa) on a Light Cycler LC480II according to manufacturer’s instructions (Roche, Switzerland). Each pool of libraries was sequenced on one flowcell of an Illumina MiSeq with 2 × 300 bp paired-end sequencing using v3 chemistry (Illumina, California, USA).

### Informatics processing

All raw reads were processed simultaneously. Fastq files were trimmed for the presence of Illumina adapters using Cutadapt^85^ version 1.2.1. 16S rRNA gene amplification primers were removed from each fragment using Trim Galore v 0.4^86^ to account for the presence of degenerate bases that might impact downstream taxonomic assignment. Reads were further trimmed using Sickle v1.33^87^ with a minimum window quality score of 28. Reads shorter than 100 bp after trimming were removed. If only one of a read-pair passed this filter, its read-pair was removed from the dataset. Surviving read pairs were merged using Pandaseq v2.9^88^ generating contigs with a minimum overlap of at least 20 bp. Merged reads greater than 450 bp were taken forward for taxonomic classification.

### Species level taxonomic assessment

Species level classification was carried out at The Forsyth Institute using an in-house pipeline. Briefly, unique assembled reads were compared to three reference databases using ncbi-blast-2.2.30+ in a sequential manner. For a successful result to be taken, sequences had to match the reference at 99% similarity across 98% of the amplicon. Reads not assigned to species level against these three databases were discarded. The databases used were the Human Oral Microbiome Database (HOMD), HOMD extended (HOMDEXT) and GreenGenes Gold, each of which had been curated to facilitate accurate species level assignment^59^.

### Statistical Analysis

Statistical analyses were performed on the table of counts produced from the bioinformatics pipeline. Counts were analysed at genus and species level, corrected for heteroscedasticity and unequal library sizes. It is common practice to employ a rarefaction process to correct for these variances in microbiome datasets^54^. However, this has been shown to be statistically inadmissible^89^. Our approach was to deal with the normalisation dependent on the context being examined. A variance stabilising transformation (VST)^89^ was applied prior to ordination using a random forest dissimilarity measure^90^,^91^,^92^. Alternatively, model-based approaches were employed for comparative testing of relative abundance between groups which explicitly model the uneven sample read numbers and sparseness. Analysis of variance (ANOVA) was conducted in parallel to determine the statistical significance using a between sample distance measure (Canberra Distance) based on taxonomic profile^93^,^94^. Analyses were performed using the R packages Vegan^95^, Party^96^ and HMP^60^.

### Visualisation

Visual exploration of trends in the samples at species level was accomplished using a novel visualisation tool MicrobiVis^97^. The bespoke MicrobiVis tool provides functionality for interactive visual analysis of microbial communities and enables interactive selection of subsets of species for in-depth detailed investigation. The selected subset of species is displayed using a Parallel Coordinates plot^98^, where each vertical axis represents one species. The individual samples are displayed as polylines that intersect the axes at positions corresponding to their microbial count for that particular species, with minimum count at the bottom of the axes and maximum count at the top. The samples at baseline were coloured blue and the samples at 14-weeks were coloured green. This approach provides an overview of the microbial profiles of the samples within the subset of species, while also facilitating the identification of outlier samples that do not follow the general profile trends. Additionally, the median profiles of the two sample groups are represented by thicker lines to provide overview of the general profile patterns of the groups. Whilst the distribution differences for individual species may be visualized using, for instance, box plots, the approach taken here has the ability to represent profiles across a group/set of species, and through this reveal sample clusters and correlations between species in more detail.

## Additional Information

**How to cite this article:** Adams, S. E. *et al*. A randomised clinical study to determine the effect of a toothpaste containing enzymes and proteins on plaque oral microbiome ecology. *Sci. Rep.* **7**, 43344; doi: 10.1038/srep43344 (2017).

**Publisher's note:** Springer Nature remains neutral with regard to jurisdictional claims in published maps and institutional affiliations.

## Supplementary Material

Supplementary Information

## References

[b1] Cho I. & Blaser M. J. The Human Microbiome: at the interface of health and disease. Nature reviews. Genetics 13, 260–270 (2012). 10.1038/nrg3182 PMC3418802 22411464

[b2] Kilian M., C. I., Hannig M., Marsh P. D., Meuric V., Pederson A. M. L. . The Oral Microbiome – An update for oral health care professionals. British Dental Journal (2016). 10.1038/sj.bdj.2016.865 27857087

[b3] Clavaud C. . Dandruff is associated with disequilibrium in the proportion of the major bacterial and fungal populations colonizing the scalp. PloS one 8, e58203 (2013). 23483996 10.1371/journal.pone.0058203 PMC3590157

[b4] Grice E. A. & Segre J. A. The skin microbiome. Nature Reviews Microbiology 9, 244–253 (2011). 21407241 10.1038/nrmicro2537 PMC3535073

[b5] Shreiner A. B., Kao J. Y. & Young V. B. The gut microbiome in health and in disease. Current opinion in gastroenterology 31, 69 (2015). 25394236 10.1097/MOG.0000000000000139 PMC4290017

[b6] Dewhirst F. E. . The Human Oral Microbiome. Journal of Bacteriology 192, 5002–5017 (2010). 20656903 10.1128/JB.00542-10 PMC2944498

[b7] Piérard‐Franchimont C., Xhauflaire‐Uhoda E. & Piérard G. Revisiting dandruff. International journal of cosmetic science 28, 311–318 (2006). 18489295 10.1111/j.1467-2494.2006.00326.x

[b8] Fitz-Gibbon S. . Propionibacterium acnes strain populations in the human skin microbiome associated with acne. Journal of Investigative Dermatology 133, 2152–2160 (2013). 23337890 10.1038/jid.2013.21 PMC3745799

[b9] Kostic A. D., Xavier R. J. & Gevers D. The microbiome in inflammatory bowel disease: current status and the future ahead. Gastroenterology 146, 1489–1499 (2014). 24560869 10.1053/j.gastro.2014.02.009 PMC4034132

[b10] Costalonga M. & Herzberg M. C. The oral microbiome and the immunobiology of periodontal disease and caries. Immunology letters 162, 22–38 (2014). 25447398 10.1016/j.imlet.2014.08.017 PMC4346134

[b11] Zarco M., Vess T. & Ginsburg G. The oral microbiome in health and disease and the potential impact on personalized dental medicine. Oral diseases 18, 109–120 (2012). 21902769 10.1111/j.1601-0825.2011.01851.x

[b12] Wade W. G. The oral microbiome in health and disease. Pharmacological research 69, 137–143 (2013). 23201354 10.1016/j.phrs.2012.11.006

[b13] Zaura E., Nicu E. A., Krom B. P. & Keijser B. Acquiring and maintaining a normal oral microbiome: current perspective Cell Infect Microbiol 4 (2014). 10.3389/fcimb.2014.00085 PMC4071637 25019064

[b14] Aas J. A., Paster B. J., Stokes L. N., Olsen I. & Dewhirst F. E. Defining the normal bacterial flora of the oral cavity. J Clin Microbiol 43, 5721–32 (2005). 16272510 10.1128/JCM.43.11.5721-5732.2005 PMC1287824

[b15] Meyle J. & Chapple I. Molecular aspects of the pathogenesis of periodontitis. Periodontology 2000 69, 7–17 (2015). 26252398 10.1111/prd.12104

[b16] Abusleme L. . The subgingival microbiome in health and periodontitis and its relationship with community biomass and inflammation. The ISME journal 7, 1016–1025 (2013). 23303375 10.1038/ismej.2012.174 PMC3635234

[b17] Hajishengallis G. The inflammophilic character of the periodontitis‐associated microbiota. Molecular oral microbiology 29, 248–257 (2014). 24976068 10.1111/omi.12065 PMC4232466

[b18] van’t Hof W., Veerman E. C., Nieuw Amerongen A. & Ligtenberg A. J. In Saliva: Secretion and Functions 40–51 (Karger Publishers, 2014).

[b19] Marsh P. D., Head D. A. & Devine D. A. Ecological approaches to oral biofilms: control without killing. Caries research 49, 46–54 (2015). 25871418 10.1159/000377732

[b20] Marsh P. D., Head D. A. & Devine D. A. Prospects of oral disease control in the future–an opinion. Journal of oral microbiology 6 (2014). 10.3402/jom.v6.26176 PMC4247391 25432790

[b21] Mandel I. D. The role of saliva in maintaining oral homeostasis. The Journal of the American Dental Association 119, 298–304 (1989). 2671090 10.14219/jada.archive.1989.0211

[b22] Dawes C. . The functions of human saliva: A review sponsored by the World Workshop on Oral Medicine VI. Archives of oral biology 60, 863–874 (2015). 25841068 10.1016/j.archoralbio.2015.03.004

[b23] Marsh P. D. & M. M. Oral Microbiology (Oxford, 1999).

[b24] de Almeida P. D. V., Gregio A., Machado M., De Lima A. & Azevedo L. R. Saliva composition and functions: a comprehensive review. J Contemp Dent Pract 9, 72–80 (2008). 18335122

[b25] Marsh P. D., Do T., Beighton D. & Devine D. A. Influence of saliva on the oral microbiota. Periodontology 2000 70, 80–92 (2016). 26662484 10.1111/prd.12098

[b26] van’t Hof W., Veerman E. C., Nieuw Amerongen A. & Ligtenberg A. J. In Saliva: Secretion and Functions 30–39 (Karger Publishers, 2014).

[b27] Hannig M. & Joiner A. In The teeth and their environment (ed. RM D.) 29–64 (Karger Publishers, Monogr Oral Sci, 2006). 10.1159/000090585 16374028

[b28] Hannig C., M. H. & Attin T. Enzymes in the acquired enamel pellicle. Eur J ORal Sci 113, 2–13 (2005). 15693823 10.1111/j.1600-0722.2004.00180.x

[b29] Marsh P. Dental plaque as a microbial biofilm. Caries research 38, 204–211 (2004). 15153690 10.1159/000077756

[b30] Tenovuo J. & Pruitt K. Relationship of the human salivary peroxidase system to oral health. Journal of Oral Pathology & Medicine 13, 573–584 (1984). 10.1111/j.1600-0714.1984.tb01459.x 6097657

[b31] Midda M. & Cooksey M. Clinical uses of an enzyme‐containing dentifrice. Journal of clinical periodontology 13, 950–956 (1986). 3098804 10.1111/j.1600-051x.1986.tb01433.x

[b32] Tenovuo J., Lumikari M. & Soukka T. Salivary lysozyme, lactoferrin and peroxidases: antibacterial effects on cariogenic bacteria and clinical applications in preventive dentistry. Proceedings of the Finnish Dental Society. Suomen Hammaslaakariseuran toimituksia 87, 197–208 (1990). 1896432

[b33] Garcia-Mendoza A., Liebana J., Castillo A. M., de la Higuera A. & Piedrola G. Evaluation of the capacity of oral streptococci to produce hydrogen peroxide. Journal of medical microbiology 39, 434–439 (1993). 8246261 10.1099/00222615-39-6-434

[b34] Thomas E. L., Milligan T. W., Joyner R. E. & Jefferson M. M. Antibacterial activity of hydrogen peroxide and the lactoperoxidase-hydrogen peroxide-thiocyanate system against oral streptococci. Infection and immunity 62, 529–535 (1994). 8300211 10.1128/iai.62.2.529-535.1994 PMC186138

[b35] Ryan C. & Kleinberg I. Bacteria in human mouths involved in the production and utilization of hydrogen peroxide. Archives of oral biology 40, 753–763 (1995). 7487577 10.1016/0003-9969(95)00029-o

[b36] Carlsson J., Edlund M. B. & Hänström L. Bactericidal and cytotoxic effects of hypothiocyanite-hydrogen peroxide mixtures. Infection and Immunity 44, 581–586 (1984). 6724690 10.1128/iai.44.3.581-586.1984 PMC263633

[b37] Hoogendoorn H. & Moorer W. Lactoperoxidase in the prevention of plaque accumulation, gingivitis and dental caries. I. Effect on oral streptococci and lactobacilli. Odontologisk revy 24, 355–366 (1972). 4522274

[b38] Hoogendoorn H., Piessens J., Scholtes W. & Stoddard L. Hypothiocyanite ion; the inhibitor formed by the system lactoperoxidase-thiocyanate-hydrogen peroxide. Caries research 11, 77–84 (1977). 265188 10.1159/000260252

[b39] Lenander-Lumikari M., Månsson-Rahemtulla B. & Rahemtulla F. Lysozyme enhances the inhibitory effects of the peroxidase system on glucose metabolism of Streptococcus mutans. Journal of dental research 71, 484–490 (1992). 1573081 10.1177/00220345920710031201

[b40] Lenander-Lumikari M., Tenovuo J. & Mikola H. Effects of a lactoperoxidase system-containing toothpaste on levels of hypothiocyanite and bacteria in saliva. Caries research 27, 285–291 (1993). 8402803 10.1159/000261552

[b41] Fadel M. & Courtois P. Effect of peroxidase-generated hypothiocyanite on the survival rate of Porphyromonas gingivalis NCTC 11834. Medical science research 27, 667–669 (1999).

[b42] Schenkels L. C., Veerman E. C. & Amerongen A. V. N. Biochemical composition of human saliva in relation to other mucosal fluids. Critical reviews in oral biology & medicine 6, 161–175 (1995). 7548622 10.1177/10454411950060020501

[b43] Nikaido H. & Vaara M. Molecular basis of bacterial outer membrane permeability. Microbiological reviews 49, 1 (1985). 2580220 10.1128/mr.49.1.1-32.1985 PMC373015

[b44] Ellison R. 3rd & Giehl T. J. Killing of gram-negative bacteria by lactoferrin and lysozyme. Journal of Clinical Investigation 88, 1080 (1991). 1918365 10.1172/JCI115407 PMC295557

[b45] Farnaud S. & Evans R. W. Lactoferrin—a multifunctional protein with antimicrobial properties. Molecular immunology 40, 395–405 (2003). 14568385 10.1016/s0161-5890(03)00152-4

[b46] Fábián T. K., Hermann P., Beck A., Fejérdy P. & Fábián G. Salivary defense proteins: their network and role in innate and acquired oral immunity. International journal of molecular sciences 13, 4295–4320 (2012). 22605979 10.3390/ijms13044295 PMC3344215

[b47] Soukka T., Lumikari M. & Tenovuo J. Combined inhibitory effect of lactoferrin and lactoperoxidase system on the viability of Streptococcus mutans, serotype c. European Journal of Oral Sciences 99, 390–396 (1991). 10.1111/j.1600-0722.1991.tb01046.x 1754841

[b48] Berlutti F., Pilloni A., Pietropaoli M., Polimeni A. & Valenti P. Lactoferrin and oral diseases: current status and perspective in periodontitis. Annali di Stomatologia 2, 10–18 (2011). 22545184 PMC3314318

[b49] Brading M. G. In 47th Meeting of CED-IADR (Oral Presentation. #0027 Antalya, Turkey, 2015).

[b50] Tenovuo J., Mansson-Rahemtulla B., Pruitt K. & Arnold R. Inhibition of dental plaque acid production by the salivary lactoperoxidase antimicrobial system. Infection and immunity 34, 208–214 (1981). 7298182 10.1128/iai.34.1.208-214.1981 PMC350844

[b51] Kirstilä V., Lenander-Lumikari M. & Tenovuo J. Effects of a lactoperoxidase-system-containing toothpaste on dental plaque and whole saliva *in vivo*. Acta Odontologica Scandinavica 52, 346–353 (1994). 7887144 10.3109/00016359409029032

[b52] Shin K. . *In vitro* and *in vivo* effects of a composition containing lactoperoxidase on oral bacteria and breath odor. Journal of breath research 2, 017014 (2008). 21386158 10.1088/1752-7155/2/1/017014

[b53] Paster B. J. . Bacterial diversity in human subgingival plaque. Journal of Bacteriology 183, 3770–3783 (2001). 11371542 10.1128/JB.183.12.3770-3783.2001 PMC95255

[b54] Huang S. . Microbiota-based Signature of Gingivitis Treatments: A Randomized Study. Scientific reports 6 (2016). 10.1038/srep24705 PMC4837389 27094556

[b55] Poole A. C. . Crossover Control Study of the Effect of Personal Care Products Containing Triclosan on the Microbiome. mSphere 1, e00056–15 (2016). 27303746 10.1128/mSphere.00056-15 PMC4888890

[b56] Belda-Ferre P. . The oral metagenome in health and disease. ISME J 6, 46–56 (2012). 21716308 10.1038/ismej.2011.85 PMC3246241

[b57] Peterson S. N. . The Dental Plaque Microbiome in Health and Disease. PLoS ONE 8, e58487 (2013). 23520516 10.1371/journal.pone.0058487 PMC3592792

[b58] Chen T. . The Human Oral Microbiome Database: a web accessible resource for investigating oral microbe taxonomic and genomic information. Database: the journal of biological databases and curation 2010, baq013 (2010). 10.1093/database/baq013 PMC2911848 20624719

[b59] Al-Hebshi N. N., Nasher A. T., Idris A. M. & Chen T. Robust species taxonomy assignment algorithm for 16S rRNA NGS reads: application to oral carcinoma samples. Journal of Oral Microbiology 7, 10.3402/jom.v7.28934 (2015). PMC4590409 26426306

[b60] La Rosa P. S., D., E., Shands B. & Shannon W. D. (http://CRAN.R-project.org/package=HMP, 2013).

[b61] Storey J. D. A direct approach to false discovery rates. Journal of the Royal Statistical Society: Series B (Statistical Methodology) 64, 479–498 (2002).

[b62] Marchesi J. R. & Ravel J. The vocabulary of microbiome research: a proposal. Microbiome 3, 1 (2015). 26229597 10.1186/s40168-015-0094-5 PMC4520061

[b63] How K. Y., Song K. P. & Chan K. G. Porphyromonas gingivalis: an overview of periodontopathic pathogen below the gum line. Frontiers in microbiology 7 (2016). 10.3389/fmicb.2016.00053 PMC4746253 26903954

[b64] Griffen A. L., Becker M. R., Lyons S. R., Moeschberger M. L. & Leys E. J. Prevalence of Porphyromonas gingivalisand Periodontal Health Status. Journal of clinical microbiology 36, 3239–3242 (1998). 9774572 10.1128/jcm.36.11.3239-3242.1998 PMC105308

[b65] Griffen A. L. . Distinct and complex bacterial profiles in human periodontitis and health revealed by 16S pyrosequencing. ISME J 6, 1176–1185 (2012). 22170420 10.1038/ismej.2011.191 PMC3358035

[b66] Kumar P. . New bacterial species associated with chronic periodontitis. Journal of dental research 82, 338–344 (2003). 12709498 10.1177/154405910308200503

[b67] Fukui K., Kato N., Kato H., Watanabe K. & Tatematsu N. Incidence of Prevotella intermedia andPrevotella nigrescens Carriage among Family Members with Subclinical Periodontal Disease. Journal of clinical microbiology 37, 3141–3145 (1999). 10488167 10.1128/jcm.37.10.3141-3145.1999 PMC85513

[b68] Nadkarni M. A. . Age-dependent changes in Porphyromonas gingivalis and Prevotella species/phylotypes in healthy gingiva and inflamed/diseased sub-gingival sites. Clinical oral investigations 19, 911–919 (2015). 25106846 10.1007/s00784-014-1301-7

[b69] Marsh P. Microbiological aspects of the chemical control of plaque and gingivitis. Journal of Dental Research 71, 1431–1438 (1992). 1629460 10.1177/00220345920710071501

[b70] Bradshaw D. J., Marsh P. D., Allison C. & Schilling K. M. Effect of Oxygen, Inoculum Composition and Flow Rate on Development of Mixed-Culture Oral Biofilms. Microbiology 142, 623–629 (1996). 8868437 10.1099/13500872-142-3-623

[b71] Public Health England Identification of Neisseria species. UK standards for microbiology investigations ID6 Issue 3 (2015).

[b72] Yost S., Duran-Pinedo A. E., Teles R., Krishnan K. & Frias-Lopez J. Functional signatures of oral dysbiosis during periodontitis progression revealed by microbial metatranscriptome analysis. Genome medicine 7, 1 (2015). 25918553 10.1186/s13073-015-0153-3 PMC4410737

[b73] Lourenço T. G. B. . Microbial signature profiles of periodontally healthy and diseased patients. Journal of clinical periodontology 41, 1027–1036 (2014). 25139407 10.1111/jcpe.12302 PMC4213353

[b74] Terai T. . Screening of probiotic candidates in human oral bacteria for the prevention of dental disease. PloS one 10, e0128657 (2015). 26053410 10.1371/journal.pone.0128657 PMC4459870

[b75] Nakano M. M. & Zuber P. Strict and facultative anaerobes: medical and environmental aspects (CRC Press, 2004).

[b76] Tanner A., Maiden M., Macuch P., Murray L. & Kent R. Microbiota of health, gingivitis, and initial periodontitis. Journal of clinical periodontology 25, 85–98 (1998). 9495607 10.1111/j.1600-051x.1998.tb02414.x

[b77] Peltroche-Llacsahuanga H., Reichhart E., Schmitt W., Lütticken R. & Haase G. Investigation of infectious organisms causing pericoronitis of the mandibular third molar. Journal of Oral and Maxillofacial Surgery 58, 611–616 (2000). 10847281

[b78] Kistler J. O., Booth V., Bradshaw D. J. & Wade W. G. Bacterial Community Development in Experimental Gingivitis. PLoS ONE 8, e71227 (2013). 23967169 10.1371/journal.pone.0071227 PMC3743832

[b79] Brown J. M., Georg L. K. & Waters L. C. Laboratory Identification of Rothia dentocariosa and Its Occurrence in Human Clinical Materials. Applied Microbiology 17, 150–156 (1969). 4886858 10.1128/am.17.1.150-156.1969 PMC377629

[b80] Chng K. R. . Whole metagenome profiling reveals skin microbiome-dependent susceptibility to atopic dermatitis flare. Nature Microbiology 1, 16106 (2016). 10.1038/nmicrobiol.2016.106 27562258

[b81] Simón-Soro Á., Guillén-Navarro M. & Mira A. Metatranscriptomics reveals overall active bacterial composition in caries lesions. Journal of oral microbiology 6 (2014). 10.3402/jom.v6.25443 PMC4247497 25626770

[b82] Zaura E. & Mira A. Editorial: The oral microbiome in an ecological perspective. Frontiers in cellular and infection microbiology 5 (2015). 10.3389/fcimb.2015.00039 PMC4413847 25973398

[b83] Welch J. L. M., Rossetti B. J., Rieken C. W., Dewhirst F. E. & Borisy G. G. Biogeography of a human oral microbiome at the micron scale. Proceedings of the National Academy of Sciences 113, E791–E800 (2016). 10.1073/pnas.1522149113 PMC4760785 26811460

[b84] Walters W. A. . PrimerProspector: *de novo* design and taxonomic analysis of barcoded polymerase chain reaction primers. Bioinformatics 27, 1159–1161 (2011). 21349862 10.1093/bioinformatics/btr087 PMC3072552

[b85] Martin M. Cutadapt removes adapter sequences from high-throughput sequencing reads. EMBnet. journal 17, pp. 10–12 (2011).

[b86] Gioti A. . Genomic insights into the atopic eczema-associated skin commensal yeast Malassezia sympodialis. MBio 4, e00572–12 (2013). 23341551 10.1128/mBio.00572-12 PMC3560662

[b87] Joshi N. A. & Fass J. N. Sickle: A sliding-window, adaptive, quality-based trimming tool for FastQ files (version 1.33) availible at https://github.com/najoshi/sickle, 2011).

[b88] Masella A. P., Bartram A. K., Truszkowski J. M., Brown D. G. & Neufeld J. D. PANDAseq: paired-end assembler for illumina sequences. BMC bioinformatics 13, 1 (2012). 22333067 10.1186/1471-2105-13-31 PMC3471323

[b89] McMurdie P. J. & Holmes S. Waste not, want not: why rarefying microbiome data is inadmissible. PLoS Comput Biol 10, e1003531 (2014). 24699258 10.1371/journal.pcbi.1003531 PMC3974642

[b90] Hothorn T., Bühlmann P., Dudoit S., Molinaro A. & Van Der Laan M. J. Survival ensembles. Biostatistics 7, 355–373 (2006). 16344280 10.1093/biostatistics/kxj011

[b91] Strobl C., Boulesteix A.-L., Zeileis A. & Hothorn T. In BMC Bioinformatics 1471–2105 (2007). 10.1186/1471-2105-8-25 PMC1796903 17254353

[b92] Strobl C., Boulesteix A.-L., Kneib T., Augustin T. & Zeileis A. Conditional variable importance for random forests. BMC bioinformatics 9, 1 (2008). 18620558 10.1186/1471-2105-9-307 PMC2491635

[b93] Anderson M. J. A new method for non-parametric multivariate analysis of variance. Austral Ecology 26, 32–46 (2001).

[b94] Gower J. C. & Krzanowski W. J. Analysis of distance for structured multivariate data and extensions to multivariate analysis of variance. Journal of the Royal Statistical Society: Series C (Applied Statistics) 48, 505–519 (1999).

[b95] Oksanen J. . Vegan: Community Ecology Package. R package version 2.0-10. 2013. (2015).

[b96] Hothorn T., Buehlmann P., Dudoit S., Molinaro A. & Laan M. V. D. 355–373 (Survival Ensembles. Biostatistics, 2006). 10.1093/biostatistics/kxj011 16344280

[b97] Fernstad S. J., Johansson J., Adams S., Shaw J. & Taylor D. In Biological Data Visualization (BioVis), 2011 IEEE Symposium on 127-134 (2011).

[b98] Inselberg A. The plane with parallel coordinates. The Visual Computer 1, 69–91 (1985).

[b99] Bik E. M. . Bacterial diversity in the oral cavity of 10 healthy individuals. ISME J 4, 962–974 (2010). 20336157 10.1038/ismej.2010.30 PMC2941673

[b100] Colombo A. P. . Comparisons of subgingival microbial profiles of refractory periodontitis, severe periodontitis, and periodontal health using the human oral microbe identification microarray. J Periodontol 80, 1421–32 (2009). 19722792 10.1902/jop.2009.090185 PMC3627366

[b101] Souto R., Andrade A. F. B. d., Uzeda M. & Colombo A. P. V. Prevalence of “non-oral” pathogenic bacteria in subgingival biofilm of subjects with chronic periodontitis. Brazilian Journal of Microbiology 37, 208–215 (2006).

[b102] van Steenbergen T. J. M., Bosch-Tijhof C. J., Petit M. D. A. & Van der Velden U. Intra-familial transmission and distribution of Prevotella intermedia and Prevotella nigrescens. Journal of Periodontal Research 32, 345–350 (1997). 9210087 10.1111/j.1600-0765.1997.tb00543.x

[b103] You M., Mo S., Watt R. M. & Leung W. K. Prevalence and diversity of Synergistetes taxa in periodontal health and disease. Journal of Periodontal Research 48, 159–168 (2013). 22881378 10.1111/j.1600-0765.2012.01516.x

