# Supplementary material for: A randomised clinical study to determine the effect of a toothpaste containing enzymes and proteins on plaque oral microbiome ecology
Source: Sci Rep. 2017 Feb 27;7:43344. doi: 10.1038/srep43344 (PMC5327414; doi:10.1038/srep43344)
Supplement: Supplementary Information [file srep43344-s1.pdf]

A randomised clinical study to determine the effect of a toothpaste containing enzymes and proteins on plaque oral microbiome ecology

Adams, SE[1], Arnold D[1], Murphy B[1], Carroll P[1], Green AK[1], Smith AM[2], Marsh PD[3], Chen T[4], Marriott RE[1] and Brading MG*[1]

[1] Unilever R&D Port Sunlight, Bebington, Wirral. CH63 3JW. UK.

[2]Unilever R&D Colworth Science Park, Sharnbrook, Bedfordshire. MK44 1LQ. UK

[3] School of Dentistry, University of Leeds. LS2 9LU. UK.

[4] Forsyth Dental Institute, 245 First Street, Cambridge, MA 02142, USA.

Supplementary Material

Beta Diversity

The difference between plaque samples, between toothpastes and across assessments was visualised using non-metric multidimensional scaling (NMDS). A random forest dissimilarity measure was employed [1,2,3]. This classifies samples based on their species profiles and will separate the groups if possible. This dissimilarity produces a good rank order to distance relationship between sample groups. As the raw count table was sparse, having a wide range of library sizes, the species table was normalised prior to analysis. A variance stabilising transformation (VST) was employed which corrects for the unequal library sizes and heteroscedasticity [4].

NMDS is an ordination technique suitable to visualise microbial community beta diversity as it only uses rank information to map into the ordination space and can handle a non-linear species response, robustly finding the underlying gradient. The plot axis scales are unit less. However, the function used, metaMDS (vegan R package [6]) rotates the NMDS solution so that the largest variation in sample scores will be on the first axis and scales so that one unit corresponds to halving the community similarity.

Dissimilarity between Samples

The random forest dissimilarity matrix was generated from the normalised species table. The VST was applied using the R package DESeq2, *varianceStabilizingTransformation* [5]. The random forest model and dissimilarity was constructed using *cforest* from the R-package party [1,2,3].

A summary of fit is summarised in Table S1 and Table S2. These show the classification of the samples compared to that observed**.** Overall the classification is very good when considering the 'in bag' classifications. When trees that did not contain a given sample in its bootstrap sample are considered ('out of bag') the classification is poorer. However, the test toothpaste, 14-week samples are classified well compared to the other sample groups, emphasising a difference in taxonomic composition.

		**Control toothpaste**		**Test toothpaste**	
**Observed**		**Baseline**	**14-Weeks**	**Baseline**	**14-Weeks**
**Control toothpaste**	Baseline	43	3	2	2
	14-Weeks	0	48	0	2
**Test toothpaste**	Baseline	2	2	44	4
	14-Weeks	0	3	1	48

Table S1 - Confusion Table (In bag classification error)

		Control toothpaste		Test toothpaste	
**Observed**		**Baseline**	**14-Weeks**	**Baseline**	**14-Weeks**
**Control toothpaste**	Baseline	5	34	6	5
	14-Weeks	30	10	3	7
**Test toothpaste**	Baseline	15	9	18	10
	14-Weeks	3	10	10	29

Table S2 - Confusion Table (Out of bag classification error)

Multidimensional Scaling

Figure S1 shows the scree plot for the nonmetric fit (Stress) values against the dimension of the representation space, for the species data. The following are guidelines for the quality of the fit,

> 0.2 poor
0.10 fair
0.05 good
0.025 excellent
0.00 perfect

A two and three dimensional representation space provide a poor fit whereas a six dimensional fit is fair. However, the two and three dimensional models differentiate the sample groups providing an easy to interpret exploratory visualisation. Figure S2 shows the Shepard plot for the three dimensional representation space.

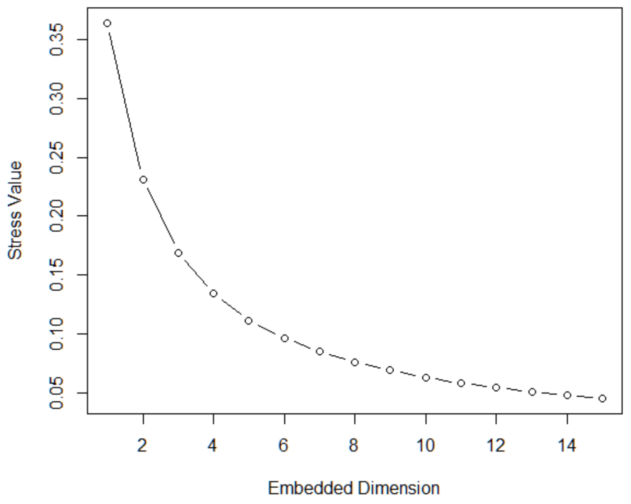

Figure S1 - Scree plot showing the stress values against embedded dimension for the random forest dissimilarity

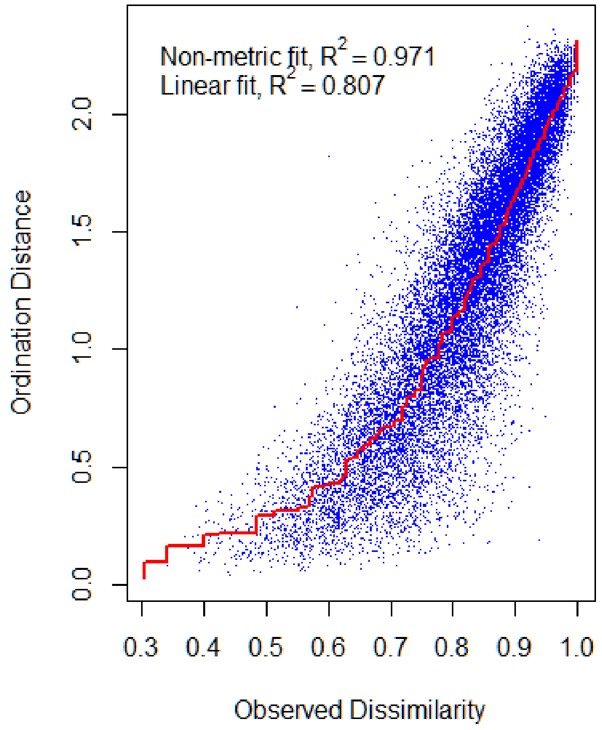

Figure S2 - Shepard Plot showing the relationship between the 3D ordered distances obtained from the random forest against the observed dissimilarities

Multivariate Analysis of Variance

The analysis was performed using a permutation based, nonparametric multivariate analysis of variance (PERMANOVA) using the *adonis* function from the R-package vegan [6]. The distance matrix was constructed using the Canberra distance. The F-tests may be biased if the dispersions within groups of samples are inconsistent [7]. These within group dispersions were compared statistically (Table S3 and Table S4) and this analysis did not provide evidence to invalidate an analysis of variance. The Canberra distance weights towards rarer species and is metric. No VST was applied as this can result in distances that are meaningless.

	Df	*Sum Sq*	*Mean Sq*	*F Value*	*Pr(>F)*
Visit*Product	3	0.004	0.0014	0.52	0.66
Residuals	200	0.534	0.0027		

Table S3- Beta Dispersion between Visit and Product groups (using the Canberra distance between samples based on genera)

	Df	*Sum Sq*	*Mean Sq*	*F Value*	*Pr(>F)*
Visit*Product	3	0.0009	0.0003	0.32	0.81
Residuals	200	0.188	0.0009		

Table S4 - Beta Dispersion between Visit and Product groups (using the Canberra distance between samples based on species)

Pre-planned contrasts between the 14-week and baseline samples for each toothpaste were tested at genus and species level. A statistically significant difference was observed for the test toothpaste but not the control. A difference in baseline diversity between the toothpastes was not supported. However, the toothpastes were statistically different at 14-weeks (not adjusted for baseline).

Genus Level

	Df	*SumsOfSqs*	*MeanSqs*	*F.Model*	*Pr(>F)*
Visit	1	0.42	0.42	1.69	0.01
Residuals	202	49.78	0.25		
Total	203	50.20			

Table S5 – Contrast between test toothpaste between baseline and 14 weeks

	Df	***SumsOfSqs***	***MeanSqs***	***F.Model***	***Pr(>F)***
Visit	1	0.17	0.17	0.70	0.97
Residuals	202	50.03	0.25		
Total	203	50.20			

Table S6 - Contrast between control toothpaste between baseline and 14 weeks

	Df	***SumsOfSqs***	***MeanSqs***	***F.Model***	***Pr(>F)***
Product	1	0.26	0.26	1.05	0.36
Residuals	202	49.94	0.25		
Total	203	50.20			

Table S7 - Comparison of toothpastes at baseline

	Df	***SumsOfSqs***	***MeanSqs***	***F.Model***	***Pr(>F)***
Product	1	0.38	0.38	0.0076	0.011
Residuals	202	49.82	0.25	0.99	
Total	203	50.20			

Table S8 - Comparison of toothpastes after 14 weeks

Species Level

	Df	***SumOfSqs***	***MeanSq***	***F.Model***	***Pr(>F)***
Visit	1	0.41	0.41	1.25	0.025
Gender	1	0.35	0.35	1.08	0.20
Residuals	202	66.24	0.33		
Total	203				

Table S9 - Contrast between test toothpaste between baseline and 14 weeks

	Df	***SumOfSqs***	***MeanSq***	***F.Model***	***Pr(>F)***
Visit	1	0.24	0.24	0.73	1.00
Gender	1	0.35	0.35	1.30	0.20
Residuals	202	66.41	0.33		
Total	203				

Table S10 - Contrast between control toothpaste between baseline and 14 weeks

	Df	***SumOfSqs***	***MeanSq***	***F.Model***	***Pr(>F)***
Product	1	0.35	0.35	1.10	0.23
Residuals	202	66.30	0.33		
Total	203				

Table S11 - Comparison of toothpastes at baseline

	Df	*SumsOfSqs*	*MeanSqs*	*F.Model*	*Pr(>F)*
Product	1	0.48	0.48	0.007	0.003
Residuals	202	66.17	0.33	0.99	
Total	203	66.65			

Table S12 - Comparison of toothpastes after 14 weeks

Differences in Mean Relative Abundance

To understand the differences observed with ordination and analysis of variance, the mean relative abundance was compared between sample groups at the species level. Tables of species count can be modelled using a multinomial distribution. However, over-dispersion is often observed. Additionally, the sampling model is such that zero counts are not truly zero and it is unlikely that all species will be observed in all samples (a multinomial model assumes a common underlying mean relative abundance vector across all samples). A hypothesis test using the C(α)-optimal test statistics of Kim and Margolin implemented in the R package HMP [8,9], tests if a multinomial distribution provides a good fit to the data compared to a Dirichlet Multinomial distribution (DM). A DM does not assume a unique underlying mean relative abundance vector, instead the counts are smoothed using the parameters of the Dirichlet. This adjusts for sparseness and unequal sample sizes. In the event a multinomial distribution provided a poor fit it was rejected in favour of the DM.

The approach taken to account for multiple test error was to control for the positive false discovery rate (pFDR) [10] using the q-value. A q-value is an adjusted p-value optimised using the characteristics of the p-value distribution generated from all the statistical tests.

Number of Subjects	Number of Subjects	Gender<br>Female/Male	Age (Years)<br>Mean; Range	
**Number of Subjects Screened**	220	143/77	42	18-75
**Subjects Accepted**	120	83/37	41	18-73
**Subjects Randomised**	115	80/35	42	18-73
**Subjects Completed**	111	78/33	42	18-73

Table S13 - Table of Study Demographics

Bibliography

[1] Torsten Hothorn, Peter Buehlmann, Sandrine Dudoit, Annette Molinaro and Mark Van Der Laan (2006). Survival Ensembles. Biostatistics, 7(3), 355--373.

[2] Carolin Strobl, Anne-Laure Boulesteix, Achim Zeileis and Torsten Hothorn (2007). Bias in Random Forest Variable Importance Measures: Illustrations, Sources and a Solution. BMC Bioinformatics, 8(25). URL http://www.biomedcentral.com/1471-2105/8/25.

[3] Carolin Strobl, Anne-Laure Boulesteix, Thomas Kneib, Thomas Augustin and Achim Zeileis (2008). Conditional Variable Importance for Random Forests. BMC Bioinformatics, 9(307). URL http://www.biomedcentral.com/1471-2105/9/307.

[4] McMurdie, Paul J., and Susan Holmes. 'Waste Not, Want Not: Why Rarefying Microbiome Data Is Inadmissible'. *PLoS Comput Biol* 10, no. 4 (3 April 2014): e1003531. doi:10.1371/journal.pcbi.1003531.

[5] Michael I Love, Wolfgang Huber and Simon Anders (2014): Moderated estimation of fold change and dispersion for RNA-Seq data with DESeq2. Genome Biology

[6] Jari Oksanen, F. Guillaume Blanchet, Roeland Kindt, Pierre Legendre, Peter, R. Minchin, R. B. O'Hara, Gavin L. Simpson, Peter Solymos, M. Henry H. Stevens and Helene Wagner (2016). vegan: Community Ecology Package. R package version 2.3-4.

https://CRAN.R-project.org/package=vegan

[7] Anderson, Marti J. ‘Distance-Based Tests for Homogeneity of Multivariate Dispersions’. *Biometrics* 62, no. 1 (March 2006): 245–53. doi:10.1111/j.1541-0420.2005.00440.x.

[8] La Rosa, PS, JP Brooks, E Deych, EL Boone, and DJ Edwards. ‘Hypothesis Testing and Power Calculations for Taxonomic-Based Human Microbiome Data’. *PLoS ONE* 7, no. 12 (2012).

[9] Patricio S. La Rosa, Elena Deych, Berkley Shands and William D. Shannon (2016). HMP: Hypothesis Testing and Power Calculations for Comparing Metagenomic Samples from HMP. R package version 1.4.3. https://CRAN.R-project.org/package=HMP

[10] Storey, John D, John Storey, and John. ‘A Direct Approach to False Discovery Rates’. *Journal of the Royal Statistical Society. Series B, Statistical Methodology* 64, no. 3 (2002): 479–98.

[11] R Core Team (2015). R: A language and environment for statistical computing. R Foundation for Statistical Computing, Vienna, Austria. URL https://www.R-project.org/.
